# The importance of friends and family to recreational gambling, at-risk gambling, and problem gambling

**DOI:** 10.1186/s12889-018-5988-2

**Published:** 2018-08-30

**Authors:** Alissa Mazar, Robert J. Williams, Edward J. Stanek, Martha Zorn, Rachel A. Volberg

**Affiliations:** 1 Social and Economic Impacts of Gambling in Massachusetts project, University of Massachusetts Amherst, School of Public Health and Health Sciences, 429 Arnold House, 715 North Pleasant Street, Amherst, MA 01003-9304 USA; 2 Faculty of Health Sciences, University of Lethbridge, M3017 Markin Hall, Alberta, T1K 3M4 Canada; 3 Social and Economic Impacts of Gambling in Massachusetts project, Amherst, MA USA; 4 Social and Economic Impacts of Gambling in Massachusetts project, University of Massachusetts Amherst, School of Public Health and Health Sciences, 418 Arnold House, 715 North Pleasant Street, Amherst, MA 01003-9304 USA

**Keywords:** Gambling, Social Networks, Recreational Gambling, Problem Gambling

## Abstract

**Background:**

The variables correlated with problem gambling are routinely assessed and fairly well established. However, problem gamblers were all ‘at-risk’ and ‘recreational’ gamblers at some point. Thus, it is instructive from a prevention perspective to also understand the variables which discriminate between recreational gambling and at-risk gambling and whether they are similar or different to the ones correlated with problem gambling. This is the purpose of the present study.

**Method:**

Between September 2013 to May 2014, a representative sample of 9,523 Massachusetts adults was administered a comprehensive survey of their past year gambling behavior and problem gambling symptomatology. Based on responses to the Problem and Pathological Gambling Measure, respondents were categorized as Non-Gamblers (2,523), Recreational Gamblers (6,271), At-Risk Gamblers (600), or Problem/Pathological Gamblers (129). With the reference category of Recreational Gambler, a series of binary logistic regressions were conducted to identify the demographic, health, and gambling related variables that differentiated Recreational Gamblers from Non-Gamblers, At-Risk-Gamblers, and Problem/Pathological Gamblers.

**Results:**

The strongest discriminator of being a Non-Gambler rather than a Recreational Gambler was having a lower portion of friends and family that were regular gamblers. Compared to Recreational Gamblers, At-Risk Gamblers were more likely to: gamble at casinos; play the instant and daily lottery; be male; gamble online; and be born outside the United States. Compared to Recreational Gamblers, Problem and Pathological Gamblers were more likely to: play the daily lottery; be Black; gamble at casinos; be male; gamble online; and play the instant lottery. Importantly, having a greater portion of friends and family who were regular gamblers was the second strongest correlate of being both an At-Risk Gambler and Problem/Pathological Gambler.

**Conclusions:**

These analyses offer an examination of the similarities and differences between gambling subtypes. An important finding throughout the analyses is that the gambling involvement of family and friends is strongly related to Recreational Gambling, At-Risk Gambling, and Problem/Pathological Gambling. This suggests that targeting the social networks of heavily involved Recreational Gamblers and At-Risk Gamblers (in addition to Problem/Pathological Gamblers) could be an important focus of efforts in problem gambling prevention.

## Background

Throughout the United States—and the world—governments are expanding the gambling options available to their populations. With this expansion, there is much interest in understanding the correlates of excessive or problem gambling. However, like the concept of gambling related harm [1], gambling behavior is not dichotomous (i.e., problem versus non-problem gambling). Instead, gambling behavior exists on a continuum. Investigating the similarities and differences between gambling subtypes allows for a more nuanced and targeted approach to reducing gambling related harm. Yet, the literature addressing which variables are related to recreational gambling or to at-risk gambling is relatively sparse [2–4] and focuses primarily on special populations, such as under-age youth [5–8] and seniors [9]. The purpose of the present study is to contribute to the limited understanding of the variables which discriminate between recreational gambling and at-risk gambling and whether they are similar or different to the ones correlated with problem gambling.

In 2011, casinos were sanctioned in Massachusetts. In anticipation of the development of casinos, a baseline measure of the gambling behavior of Massachusetts residents was collected in 2013-2014. This analysis uses this baseline measure—the Baseline General Population Survey (BGPS) of Massachusetts—which contains a representative sample of 9,523 Massachusetts adults who were administered a comprehensive survey of their past year gambling behavior and problem gambling symptomology. Individuals’ gambling behavior were classified based on the Problem and Pathological Gambling Measure (PPGM) [10, 11]—which offers a sophisticated and sensitive measure of gambling subtypes—to identify variables related to gambling involvement. By teasing out the discriminative differences between gambling subtypes, these analyses provide: 1) directions for targeted interventions to reduce gambling related harm and 2) a baseline understanding of gambling behavior prior to the development of casinos in Massachusetts.

### Discriminators of Gambling Category

#### Non-Gamblers

Using All Gamblers as the comparative group, studies examining the attributes of Non-Gamblers have found the following variables correlated to being a Non-Gambler: female gender [12, 13]; ethnic/racial group (e.g., African-Americans in the U.S.) [14, 15]; age (both younger and older people) [12, 13, 15]; lower socioeconomic status [13, 15]; and higher educational attainment [13]. Based on this research, it is clear that there are significant differences between Non-Gamblers and All-Gamblers. Unlike previous research, our analysis compares the differences between more fine-grained subtypes of gamblers which are less explored and potentially more similar: Non-Gamblers and Recreational Gamblers. These (dis)similarities are relevant to whether prevention of gambling related harm should be directed at gambling in general or should be more targeted toward excessive gambling.

#### At-Risk Gamblers

Longitudinal studies have found that at-risk gambling is one of the strongest predictors of future problem gambling [16–18]. In addition, since the size of the At-Risk category is generally larger than the Problem Gambler category, At-Risk Gamblers may also represent a larger burden on society. Despite this, the existing literature on correlates of at-risk gambling is surprisingly limited.

Using a 2002 Norwegian national gambling survey totaling 4,188 respondents, Lund [17] finds that At-Risk Gamblers (who have experienced one or two negative consequences in the last 12 months) differ from No-Risk Gamblers (who have not experienced any negative consequences in the last 12 months). At-Risk Gamblers are more likely to be men, young people, divorced or single people, and non-western immigrants. Furthermore, At-Risk Gamblers are more likely to have gambling problems in the family. Using a 2005 Danish survey of 4,392 current gamblers with no gambling problem or pathology and a second wave re-interviewing 379 respondents in 2006, Lyk-Jensen [19] finds that at-risk gambling is more prevalent among men, young-to-middle-aged people, and immigrants. At-Risk Gamblers are also more likely than No-Risk Gamblers to have low income and low education. Interestingly, Lyk-Jensen [19] finds that high stakes gambling among acquaintances (friends and colleagues at work or school) and family also increases the likelihood of at-risk gambling in this study.

#### Problem Gamblers

Identification of variables associated with problem gambling has obvious implications for prevention and treatment. Not surprisingly, there have been many studies that have identified cross-sectional and/or longitudinal correlates of problem gambling, including the characteristics and behaviors of Problem Gamblers. These include, for example: male gender [20, 21]; non-Caucasian or a member of a minority group [21, 22]; young age (18 – 25) [20, 21]; less education [16, 23]; being divorced or separated [24, 25]; lower income [21, 26]; family history of gambling and/or problem gambling [27, 28]; peer group or friends involvement in gambling [18, 29]; poorer physical health [30, 31]; substance use and abuse [32–34]; greater intensity of gambling involvement as measured by higher frequency, expenditure, and number of formats engaged in [18, 35]; engaging in ‘continuous’ forms of gambling (electronic gambling machines) that provide a high frequency of reinforcement [36, 37]; and internet gambling [38, 39].

As described above, there is an immense list of variables which discriminate between gambling subtype behavior. It is also evident that demographic, social, and gambling related variables correlate to gambling behavior and gambling related harm; yet *how much* these variables matter—or which offer the strongest discriminative power—is dependent on the particulars of the population being considered. Therefore, rather than picking particular hypotheses to test, we chose to allow the data ‘to speak’ to this Massachusetts sample and then discuss how these findings relate to the broader body of research.

Unlike previous analyses, the present analysis looks across four types of gambling participation: Non-Gamblers, Recreational Gamblers, At-Risk Gamblers, and Problem/Pathological Gamblers. This analysis also uses the comparative group of Recreational Gambler, which is the most common form of gambling behavior. Furthermore, by utilizing the PPGM to classify respondents—which offers a holistic measure to accurately capture the spectrum of gambling behavior—this study provides insights into the complexity of gambling behavior to inform prevention and treatment to reduce gambling related harm.

## Methods

The Baseline General Population Survey (BGPS) of Massachusetts was conducted by NORC at the University of Chicago under contract to the University of Massachusetts Amherst, School of Public Health and Health Sciences. The goals of the study were to establish a baseline level of gambling participation and problem gambling prevalence and to assess awareness and utilization of problem gambling services prior to the opening of new gambling facilities in Massachusetts. The survey protocol was reviewed and approved separately by NORC’s Institutional Review Board and by the University of Massachusetts Amherst Institutional Review Board. The study was also subject to independent peer review by the Massachusetts Gaming Commission’s Gaming Research Advisory Committee.

The BGPS used address-based probability sampling to ensure that all Massachusetts households had a known probability of selection into the sample. Within each sampled dwelling unit, the adult with the most recent birthday was selected as the survey respondent. Data collection began in September 2013 and ended in May 2014. The response rate was 36.6% and the final sample included 9,578 Massachusetts residents aged 18 and over. NORC mailed letters to all selected addresses and subsequent postcards inviting the adult (18+) household member with the most recent birthday to complete an online survey. The letter contained a $1 incentive and offered respondents a $10 Amazon gift-code if the survey was completed within 14 days. A thank-you or reminder postcard was mailed out one week after the advance letter. Two weeks later, a second postcard was mailed out. If respondents had not completed the survey online four weeks after the advance letter, they were sent a paper-and-pencil questionnaire along with an explanatory letter, a $5 incentive, and a return envelope. Two weeks later, a thank-you or reminder postcard was mailed out. Two weeks later, households received a second invitation letter along with a second copy of the questionnaire. Every address that failed to complete the survey via mail or online and whose household had been matched with a landline telephone number was then called and given the opportunity to complete the survey over the telephone. Telephone interviews were conducted by trained interviewers using a CATI system. The majority of questionnaires were self-administered, with 40% completed online and 52% completed using the paper-and-pencil questionnaire. A total of 152 questionnaires or telephone interviews (1.6%) were completed in Spanish.

### Questionnaire

The questionnaire included sections on recreation, physical, and mental health behaviors, alcohol and drug use, attitudes toward gambling, gambling participation, gambling motivations, awareness of problem gambling services, gambling-related problems, and demographics. Gambling participation was assessed by asking about past year frequency of participation in 11 different types of gambling: lottery tickets; instant tickets or pull tabs; daily lottery games; raffle tickets; betting money on sporting events (this includes sports pools); bingo; casino, racino, or slots parlor outside of Massachusetts; horse racing (on-site track or an off-track site); betting money against other people on things such as card games, golf, pool, darts, bowling, video games, board games, or poker outside of a casino; high risk stocks, options, or futures or day trade on the stock market; and gambling online, which includes playing poker, buying lottery tickets, betting on sports, bingo, slots or casino table games for money, or playing interactive games for money.

All participants who reported gambling once a month or more on some type of gambling were administered the PPGM. The PPGM was developed to rectify the weak correspondence between Problem and Pathological Gamblers identified in population surveys and subsequent classification of these individuals in clinical interviews. The PPGM has higher sensitivity, specificity, and classification accuracy compared to older instruments such as the Canadian Problem Gambling Index (CPGI), Diagnostic and Statistical Manual of Mental Disorders—Fourth Edition (DSM-IV), and the South Oaks Gambling Screen (SOGS) (For additional information on the PPGM see [10, 11]).

Based on responses to the PPGM, a person was categorized as a Non-Gambler if he or she reported no past year participation in any form of gambling (with the exception of high-risk stocks). 2,523 respondents (26.5%) received this classification. A person was categorized as a Recreational Gambler if he or she reported participating in one or more types of gambling in the past year but no problem gambling symptomatology and frequency of gambling and gambling expenditure were below levels reported by Problem and Pathological Gamblers. 6,271 respondents (65.9%) received this classification. A person was categorized as an At-Risk Gambler if he or she reported participating in one or more types of gambling in the past year *and* reported one or more symptoms of problem gambling. Alternatively, a person could be classified as an At-Risk Gambler if their frequency of gambling and gambling losses were equal to or greater than the median reported for Problem and Pathological Gamblers. A total of 600 respondents (6.3%) received this classification. A person was categorized as a Problem Gambler if he or she reported: gambling at least once a month on one or more types of gambling; a Problems Score of 1 or higher; an Impaired Control Score of 1 or higher; and a Total Score of 2 to 4. Alternatively, a person could receive this designation if they had a Total Score of 3 or higher plus a frequency of gambling and reported gambling loss that was equal to or greater than the median for Problem and Pathological Gamblers. 75 respondents (0.79%) received this classification. A person was categorized as a Pathological Gambler if he or she reported: gambling at least once a month on one or more types of gambling; a Problems Score of 1 or higher; an Impaired Control Score of 1 or higher; and a Total Score of 5 or higher. 54 people (0.57%) received this classification. In the statistical analyses, Problem and Pathological Gamblers were collapsed into one group due to small cell size. Table 1 displays the race/ethnicity and gender of the achieved sample of respondents by gambling behavior.

**Table 1:** Race/Ethnicity and Gender of Baseline General Population Survey Respondents by Gambling Behavior*

Race/Ethnicity	Gender	Non- Gambler	Recreational Gambler	At-Risk Gambler	Problem Gambler/Pathological Gambler
White	Male	681	2156	258	65
	Female	1260	3208	221	30
	Missing	9	35	*	
Hispanic	Male	48	86	17	*
	Female	120	173	19	*
	Missing	*		*	
Black	Male	39	64	18	13
	Female	74	123	23	5
	Missing		*		*
Asian	Male	68	82	17	*
	Female	101	83	6	*
	Missing	*			
Other	Male	7	20	*	
	Female	22	26	*	
	Missing	*	*		
Missing	Male	36	95	9	
	Female	35	89	*	*
	Missing	18	29	*	
TOTAL		2523	6271	600	129

*These cells do not report a frequency due to small cell sizes

### Statistics

With the reference category of Recreational Gambler, multivariate logistic regressions included demographic, health, and gambling related variables. Thirty-two independent variables (Table 2) were examined to determine whether there were significant differences between: a) Recreational and Non-Gamblers, b) Recreational and At-Risk Gamblers, and c) Recreational and Problem/Pathological Gamblers. Unweighted data were used in all of the analyses since the focus was on identifying differences or relationships within the data, independent of the data’s relationship to the general population.

**Table 2:** Independent variables

Demographic	Health-related	Gambling-related
1. gender	1. health status in past 12 months	1. portion of friends and family that are regular gamblers
2. age	2. participation in extreme sports	2. lottery purchase in the past 12 months
3. race/ethnicity	3. overall level of stress in the past 12 months	3. daily lottery purchase in the past 12 months
4. born in the United States	4. current tobacco use	4. instant lottery purchase in the past 12 months
5. marital status	5. alcohol use in the past 30 days	5. raffle purchase in the past 12 months
6. educational attainment	6. binge drinking in the past 30 days	6. sports betting in the past 12 months
7. employment	7. illicit drug use in the past 12 months	7. bingo participation in the past 12 months
8. household income	8. drug or alcohol problems in the past 12 months	8. horse race betting in the past 12 months
9. military service	9. behavioral addictions in the past 12 months (overeating, sex or pornography, shopping, exercise, Internet chat lines, etc.)	9. private betting in the past 12 months
10. geographic region of residence in Massachusetts	10. serious mental health problems in the past 12 months	10. casino gambling in the past 12 months
	11. rating of childhood happiness	11. online gambling in the past 12 months

Eleven gambling-related variables were used in the comparisons between Recreational and At-Risk Gamblers and Recreational and Problem/Pathological Gamblers. Only one gambling related variable was used when comparing Recreational and Non-Gamblers, which asked about the portion of friends and family who gambled regularly. The other gambling related variables required that the individual gamble and therefore were not applicable for that comparison.

Allowing various gambling formats to enter into the models is important for two reasons. First, it has been demonstrated that different gambling formats are correlated differently to gambling behavior and pose differing levels of risk for the gambler. For example, the likelihood of casino gambling (which includes EGMs and tables games) resulting in gambling harm is much higher than playing traditional lottery games. Second, demonstrating the discriminative differences between gambling formats and gambling categorization has important policy implications as new forms of gambling are legalized and their availability expands. The strong relationship between problem gambling and engaging in certain forms of gambling (e.g., online gambling), however, is partly due to the fact that certain types of gambling are engaged in by individuals with high levels of gambling involvement [40, 41]. Supplemental analyses were undertaken to examine the contribution of individual forms of gambling to at-risk gambling and problem/pathological gambling after controlling for the number of gambling formats engaged in.

Binary logistic regressions were performed for all variables collectively. Variables entered into the logistic regression in a forward stepwise manner, with variable entry order determined by the size of the Wald statistic (minimum entry level of *p* = .01 and a removal level of *p* = .05). The Wald statistic assesses the statistical significance of the coefficients. It is analogous to the t-test for assessing the significance of a coefficient in a bivariate correlation. Missing values were replaced using multiple imputation. This involved imputing values for the 11 variables having the greatest number of missing values (i.e., household income, casino participation, mental health problems, age, binge drinking, race/ethnicity, marital status, being born in the United States, employment status, educational attainment, and current tobacco use) using a multivariate model that predicted a set of 10 likely values using the 25 variables having the strongest univariate association to the 11 aforementioned variables.

The percentage of missing data in the imputed variables was from 2%-7% for casino participation, mental health problems, age, binge drinking, race/ethnicity, marital status, being born in the US, employment status, education attainment, and current tobacco use and 14% for household income. The justification for imputing this data is that only 66% of the 9,758 respondents in the sample would have been available for analysis under the traditional listwise deletion method. We also did not cut individuals that had a given percentage of missing data. Analyses were run for each of the imputed datasets and the results of these 10 imputations were then pooled using Rubin’s rule [42] to account for variability incurred through introduction of the imputed data. Relative efficiency was close to 1.0 for all 11 variables, indicating that the 10 imputations were sufficient. Recreational Gambler was used as the reference group to provide consistency across analyses and because recreational gambling is the normative/modal gambling category in Massachusetts, as it is in other jurisdictions.

## Results

A binary logistic regression found maximal discrimination between Recreational and Non-Gamblers via a model with a constant and 13 correlates. Table 3 shows the log of the odds ratio and Wald statistic for each of the 13 correlates. The variance accounted for was low with an adjusted *R* squared ranging from 12.2% to 12.6% (depending on the imputation). Using a classification cutoff of 28.0% to maximize both sensitivity and specificity, overall prediction success ranged from 62.1% to 62.9%. In order of importance, people who were Non-Gamblers were significantly more likely than Recreational Gamblers to: have a lower portion of friends and family that were regular gamblers; not use alcohol; have higher educational attainment; be a student, homemaker, disabled, or retired; be either 18-34 or 65+; be born outside the United States; not binge drink; have lower household income; not use tobacco; have less happy childhoods; not have served in the military; not have problems with drugs or alcohol; and be non-White.

**Table 3:** Stepwise Logistic Regression Predicting for Non-Gambling compared to Recreational Gambling (*n* = 8,794)

	Odds Ratio & 95% C.I.	Wald Statistics	*p*
Portion of Friends and Family Regular Gamblers	.64 (0.59, 0.71)	89.2	< .0001
Alcohol use in Past 30 Days			
No	1.72 (1.53, 1.93)	85.5	< .0001
Yes	Reference group		
Education			
High School or Less	Reference group	Reference group	
Bachelor’s or some College	1.07 (0.93, 1.23)	0.9	.0029
Beyond Bachelor’s degree	1.72 (1.46, 2.03)	41.3	< .0001
Employment			
Employed	Reference group	Reference group	
Unemployed	1.00 (0.75, 1.33)	0.0	.8811
Retired	1.17 (0.98, 1.38)	3.1	< .0001
Other^a^	1.68 (1.43, 1.97)	41.1	< .0001
Age			
35-64	Reference group	Reference group	
18-34	1.60 (1.37, 1.86)	38.2	< .0001
65+	1.34 (1.14, 1.57)	12.4	< .0001
Born in United States			
No	1.57 (1.33, 1.85)	28.3	< .0001
Yes	Reference group		
Binge Drinking			
No	1.43 (1.24, 1.65)	25.3	< .0001
Yes	Reference group		
Household Income	.97 (0.96, 0.98)	23.4	< .0001
Current Tobacco use			
No	1.42 (1.20, 1.69)	16.9	< .0001
Yes	Reference group		
Unhappy Childhood	1.12 (1.06, 1.18)	16.8	< .0001
Military Service			
No	1.32 (1.10, 1.58)	9.0	< .0001
Yes	Reference group		
Problems with Drugs or Alcohol			
No	2.14 (1.28, 3.57)	8.5	< .0001
Yes	Reference group		
Race/Ethnicity			
White	Reference	Reference	
Hispanic	1.19 (0.94, 1.51)	2.1	.0048
Black	1.44 (1.11, 1.86)	7.7	<.0001
Asian	1.45 (1.10, 1.91)	8.0	.0017
Other	1.54 (0.95, 2.49)	3.2	.0001

^a^Student, homemaker, disabled were combined into ‘Other’ because of small sample sizes in each

Maximal discrimination between Recreational and At-Risk Gamblers occurred with a model including a constant and 14 correlates. Table 4 shows the log of the odds ratio and Wald statistic for each of the 14 correlates. The variance accounted for was modest with an adjusted *R* squared ranging between 21.9% and 22.0% for the 10 imputations. Using a classification cutoff of 8.0% to maximize both sensitivity and specificity, overall prediction success ranged between 70.8% and 71.0%. In order of importance, people who were At-Risk Gamblers were significantly more likely to: be a casino gambler; have a greater portion of friends and family that are regular gamblers; play instant lottery games; play daily lottery games; be male; be an online gambler; be born outside of the United States; participate in private betting; have lower educational attainment; play bingo; not purchase raffle tickets; have lower household income; have mental health problems; and have no alcohol use in the past 30 days.

**Table 4:** Stepwise Logistic Regression Predicting for At-Risk Gambling compared to Recreational Gambling (*n* = 6,871)

	Odds Ratio & 95% C.I.	Wald Statistics	*p*
Casino Gambling			
No	Reference group	110.4	< .0001
Yes	2.73 (2.26, 3.29)		
Portion of friends and family regular gamblers	2.16 (1.86, 2.51)	101.0	< .0001
Instant Lottery			
No	Reference group	48.3	< .0001
Yes	2.04 (1.67, 2.50)		
Daily Lottery Games			
No	Reference group	41.6	< .0001
Yes	1.97 (1.61, 2.43)		
Gender			
Male	1.60 (1.33, 1.94)	24.1	< .0001
Female	Reference group		
Online Gambling			
No	Reference group	22.1	< .0001
Yes	3.31 (2.01, 5.46)		
Born in United States			
No	1.79 (1.37, 2.32)	19.0	< .0001
Yes	Reference group		
Private Betting			
No	Reference group	18.3	< .0001
Yes	1.70 (1.34, 2.18)		
Education			
Beyond Bachelor’s degree	Reference group	Reference group	
High school or less	1.92 (1.40, 2.63)	16.7	< .0001
Bachelor’s or some College	1.24 (0.95, 1.62)	2.4	< .0001
Bingo			
No	Reference group	13.4	< .0001
Yes	1.88 (1.34, 2.64)		
Raffles			
No	1.30 (1.08, 1.57)	7.3	< .0001
Yes	Reference group		
Household income	.97 (0.95, 0.99)	6.9	.0008
Mental health problems past 12 months			
No	Reference group	6.7	.0002
Yes	1.36 (1.07, 1.73)		
Alcohol use past 30 days			
No	1.32 (1.07, 1.63)	6.5	< .0001
Yes	Reference group		

A supplemental analysis was undertaken to examine the contribution of individual forms of gambling to at-risk gambling status after controlling for the number of gambling formats engaged in. This was done by adding number of gambling formats as an additional predictor variable. Entering the number of gambling formats engaged in as an additional variable helps determine whether there are specific types of gambling that provide additional power to predict at-risk gambling after number of gambling formats enters the model. As seen in Table 5, when number of gambling formats is added to the model, casino gambling and non-involvement in raffles still add discriminative power. Also, as expected, number of gambling formats becomes the most powerful discriminative variable as it is best seen as an aspect of at-risk gambling.

**Table 5:** Stepwise Logistic Regression Predicting for At-Risk Gambling compared to Recreational Gambling after Controlling for Number of Gambling Formats Engaged In (n = 6,871)

	Odds Ratio & 95% C.I.	Wald Statistics	*p*
Number of gambling formats engaged in	1.63 (1.51, 1.76)	165.0	< .0001
Portion of friends and family regular gamblers	2.12 (1.82, 2.46)	96.6	< .0001
Raffles			
No	2.13 (1.71, 2.66)	46.1	< .0001
Yes	Reference group		
Born in United States			
No	1.86 (1.43, 2.42)	21.9	< .0001
Yes	Reference group		
Casino Gambling			
No	Reference group	20.3	< .0001
Yes	1.63 (1.32, 2.02)		
Education			
Beyond Bachelor’s degree	Reference group	Reference group	
High school or less	1.93 (1.41, 2.63)	17.1	< .0001
Bachelor’s or some College	1.25 (.96, 1.63)	2.7	< .0001
Gender			
Male	1.46 (1.21, 1.76)	16.1	< .0001
Female	Reference group		
Household income	.96 (.94, .99)	11.3	.0002
Mental health problems past 12 months			
No	Reference group	9.9	< .0001
Yes	1.45 (1.14, 1.83)		
Alcohol use past 30 days			
No	1.33 (1.08, 1.65)	7.3	< .0001
Yes	Reference group		

Maximal discrimination between Recreational Gamblers and Problem/Pathological Gamblers occurred for a model with a constant and 11 correlates (Table 6). The variance accounted for was again modest with an adjusted *R* squared ranging between 30.7% and 31.1%. Using a classification cutoff of 2% to maximize both sensitivity and specificity, overall prediction success was between 81.4% and 81.7%. In order of importance, people who were Problem/Pathological Gamblers were significantly more likely to: play daily lottery games; have a greater portion of friends and family that are regular gamblers; be Black; be a casino gambler; be male; be an online gambler; play instant lottery games; have behavioral addictions; have lower educational attainment; be born outside of the United States; and have less happy childhoods.

**Table 6:** Stepwise Logistic Regression Predicting for Problem and Pathological Gambling compared to Recreational Gambling (*n* = 6,400)

	Odds Ratio & 95% C.I.	Wald Statistics	*p*
Daily Lottery Games			
No	Reference group	28.2	< .0001
Yes	3.00 (2.00, 4.50)		
Portion of friends and family regular gamblers	2.25 (1.66, 3.05)	27.9	< .0001
Race/Ethnicity			
White	Reference group	Reference group	
Other	.86 (0.30, 2.43)	0.1	.8420
Hispanic	.70 (0.28, 1.79)	0.6	.3097
Black	4.60 (2.55, 8.30)	25.8	< .0001
Casino Gambling			
No	Reference group	23.1	< .0001
Yes	2.65 (1.78, 3.94)		
Gender			
Male	2.62 (1.75, 3.92)	22.1	< .0001
Female	Reference group		
Online Gambling			
No	Reference group	19.8	< .0001
Yes	5.71 (2.65, 12.30)		
Instant Lottery			
No	Reference group	15.4	< .0001
Yes	2.70 (1.64, 4.43)		
Behavioral Addictions			
No	Reference group	14.3	< .0001
Yes	2.34 (1.50, 3.65)		
Education			
Beyond Bachelor’s degree	Reference group	Reference group	
High school or less	3.27 (1.69, 6.33)	13.2	< .0001
Bachelor’s or some College	1.20 (0.63, 2.28)	0.4	.3333
Born in United States			
No	2.49 (1.42, 4.34)	10.3	< .0001
Yes	Reference group		
Unhappy Childhood	1.38 (1.12, 1.69)	9.1	< .0001

A supplemental analysis was undertaken to examine the contribution of individual forms of gambling to problem/pathological gambling status after controlling for the number of gambling formats engaged in. As shown in Table 7, when number of gambling formats is added to the model, the only type of gambling that added power in discriminating for problem or pathological gambling from recreational gambling was non-involvement in raffle tickets and engagement in private gambling. As expected, number of gambling formats becomes the most powerful predictive variable as it is best seen as a manifestation of problem/pathological gambling.

**Table 7:** Stepwise Logistic Regression Predicting for Problem and Pathological Gambling compared to Recreational Gambling after Controlling for Number of Gambling Formats Engaged In (n = 6,400)

	Odds Ratio & 95% C.I.	Wald Statistics	*p*
Number of gambling formats engaged in	2.22 (1.91, 2.57)	112.6	< .0001
Race/Ethnicity			
Other	0.90 (0.32, 2.52)	0.1	.8959
Hispanic	0.65 (0.26, 1.62)	0.9	.1712
Black	4.69 (2.59, 8.50)	26.3	< .0001
White	Reference group	Reference group	
Portion of friends and family regular gamblers	2.07 (1.53, 2.79)	22.8	< .0001
Raffles			
No	2.81 (1.76, 4.49)	18.6	< .0001
Yes	Reference group		
Gender			
Male	2.29 (1.52, 3.45)	15.7	< .0001
Female	Reference group		
Born in United States			
No	3.03 (1.73, 5.30)	15.3	< .0001
Yes	Reference group		
Education			
Beyond Bachelor’s degree	Reference group	Reference group	
High school or less	3.06 (1.57, 5.98)	11.5	< .0001
Bachelor’s or some College	1.23 (0.64, 2.36)	0.5	.2785
Beyond Bachelor’s degree	Reference group	Reference group	
Behavioral Addictions			
No	Reference group	9.8	< .0001
Yes	2.05 (1.30, 3.24)		
Childhood Unhappiness	1.37 (1.11, 1.70)	8.5	< .0001
Poorer health status	1.32 (1.08, 1.61)	7.2	< .0001
Private betting			
No	2.17 (1.23, 3.85)	7.1	< .0001
Yes	Reference group		

## Discussion

Using data from the 2013-2014 BGPS of Massachusetts adults, these analyses focus on the multivariate differences between Recreational Gamblers and three other types of gambling behavior: Non-Gamblers, At-Risk Gamblers, and Problem/Pathological Gamblers. To our knowledge, this is the first study to analyze all of the subtypes of gambling behavior within one dataset and with Recreational Gambler—the most common type of gambler—as the reference group. These analyses offer a consistent picture of the similarities and differences between gambling subtypes. This allows for better discrimination between the correlates of gambling behavior with the aim of reducing gambling related harm and promoting more efficient allocation of prevention and treatment efforts. Furthermore, by utilizing the PPGM to classify gambling behavior, this study benefits from the superior sensitivity, specificity, and classification accuracy of this instrument [10, 11].

The analysis of differences between Recreational and Non-Gamblers identifies variables that have not previously been found to be discriminative of Non-Gamblers. These are: not using alcohol or tobacco; not having problems with drugs or alcohol; having a smaller portion of friends and family that are regular gamblers; being a student, homemaker, or disabled; not having served in the military; being an immigrant; and having a somewhat lower level of childhood happiness. The strongest discriminator of being a Non-Gambler rather than a Recreational Gambler was the single gambling-related variable: having a lower portion of friends and family that are regular gamblers.

The ability of the multivariate model to discriminate between Non-Gamblers and Recreational Gamblers was relatively weak. The implication is that there are many similarities between the two groups. Many Recreational Gamblers are designated as such simply because of their occasional purchase of lottery or raffle tickets. Similarly, a portion of occasional raffle or lottery ticket purchasers would be classified as Non-Gamblers if they made no purchases in the past year.

The ability of the multivariate model to discriminate between Recreational Gamblers and At-Risk Gamblers was modest. Part of the reason for the improved discriminative ability relative to the previous analysis is the addition of gambling participation variables utilized for the At-Risk analysis. Nonetheless, the results indicate that some important demographic and health differences exist between these groups.

Demographically, At-Risk Gamblers compared to Recreational Gamblers are more likely to be male, to be born outside the United States, to have lower educational attainment, and to have a lower household income. These results are similar to Lund’s [43] and Lyk-Jensen’s [19] findings. Unlike Lund and Lyk-Jensen, age and marital status are not significantly correlated to being in the At-Risk category in the Massachusetts sample. In terms of health, At-Risk Gamblers in Massachusetts are more likely than Recreational Gamblers to have mental health problems and not to have used alcohol in the past 30 days. It is unclear why alcohol abstinence is a correlate of at-risk gambling. This could be related to the bimodal distribution of alcohol use in individuals with a history of alcohol abuse or who come from a family with alcohol abusers [44]. Like Lund [43] and Lyk-Jensen [19], the Massachusetts study also finds that family and peer groups matter in that gambling problems or high gambling involvement among family, friends, and colleagues increases the likelihood of being in the At-Risk Gambler category. More surprising was how strong of a correlate having a greater portion of family and friends who are regular gamblers was in discriminating At-Risk Gamblers from Recreational Gamblers as it was the second strongest correlate.

Unsurprisingly, the ability of the multivariate logistic model to discriminate between Recreational Gamblers and Problem/Pathological Gamblers was stronger than any of the other analyses. Demographically, Problem and Pathological Gamblers were more likely to be Black, male, have lower educational attainment, and be born outside the United States compared to Recreational Gamblers. It is worth noting that both Non-Gamblers and Problem/Pathological Gamblers in Massachusetts are significantly more likely to be non-White than Recreational Gamblers. It is possible that non-Whites in Massachusetts represent a bimodal group in the population, with a relatively large proportion who have little or no involvement in gambling and a significant minority who gamble frequently and experience gambling-related difficulties. This pattern has been found among recent immigrants, youth, and women in other jurisdictions and may reflect recent exposure to legal commercial gambling as well as heightened vulnerability to the development of gambling-related difficulties [45]. Similar to Non-Gamblers and At-Risk Gamblers, classification as a Problem/Pathological Gambler was highly related to the portion of family and friends who regularly gambled, being the second strongest correlate.

The present results also reaffirm prior research showing that certain types of gambling have a higher risk profile than other types. Casino gambling, which was the strongest individual predictor of at-risk gambling status, primarily involves slot machines and casino table games, which have a strong association to gambling-related harm because of their continuous nature [37, 46]. Instant lottery games were also a strong predictor of at-risk gambling, which may be similarly related to the short period of time between the wager and the outcome and the ability to immediately rewager [47–49]. It is important to note that although the majority of problem gamblers in Massachusetts do not identify any particular type of gambling as being more problematic than others, those that do identify a problematic format are most likely to identify instant lottery games [50]. Finally, as has also been found in previous research, online gambling was a significant predictor of at-risk gambling, presumably due to its 24-hour availability, convenience, and the fact that it offers continuous forms of gambling.

The caveat to these gambling-related results is that only casino gambling and not participating in raffles remain significant in discriminating At-Risk Gamblers from Recreational Gamblers after number of gambling formats engaged in was entered into the multivariate model. This is a further reminder that most At-Risk and Problem Gamblers engage in several different types of gambling, all of which contribute to their problems and there is often not a singular problematic format. At the same time, it is important to recognize that entering number of gambling formats into the multivariate model has significant limitations in illustrating the importance of specific gambling formats. The most important limitation is that extensive involvement in several different types of gambling is ***one aspect*** of being an At-Risk Gambler or a Problem/Pathological Gambler, which is why it is not normally used as a predictor (and why aggregate gambling frequency and total gambling expenditure were also not used as predictors). This is also why it is overwhelmingly the strongest predictor when entered into the model. When an aspect of a disorder is entered as a predictor of the disorder, it becomes very difficult for other variables to add any discriminative power as it is analogous to trying to predict Pathological Gambling after Problem Gambling is entered as a predictor or Major Depression after low mood is entered as a predictor.

The other challenge to this approach concerns the equivalency and substitutability of more harmful forms of gambling. Using a drug example, polydrug use is common among drug abusers. Some use caffeine, tobacco, and heroin; some use caffeine, tobacco, and cocaine; some use caffeine, cannabis, and methamphetamine, etc. Thus, using multiple drugs is a very strong predictor of drug abuse and it is often not possible to statistically show that heroin use, or cocaine use, or methamphetamine use have additive harm, even though it is self-evident they are causing the most problems and are what people are seeking treatment for. The issue has to do with the substitutability and equivalency of more harmful substances. In other words, the person who is using heroin is just as impaired as the person using cocaine who is just as impaired as the person using methamphetamine. It is difficult to show an addictive effect of heroin, cocaine, or methamphetamine when controlling for the number of drugs.

When examining the discriminative differences between Problem/Pathological Gamblers and Recreational Gamblers, gambling-related variables were the strongest discriminators, with the following variables significantly predicting greater likelihood of being a Problem or Pathological Gambler: playing daily lottery games; having a greater portion of friends and family involved in gambling, engaging in casino gambling, engaging in online gambling, and playing instant lottery games. However, none of these gambling formats were predictive when controlling for number of gambling formats engaged in. Private betting and non-involvement in raffles do become significant, however. As before, this finding reiterates that problem gamblers typically engage in several different types of gambling, all of which contribute to their difficulties. Yet, entering number of gambling formats into the model reduces the marginal (or incremental) importance of individual types of gambling. One interesting and unique finding not previously reported in the literature is that non-involvement in purchasing raffle tickets in Massachusetts is predictive of being an At-Risk or Problem/Pathological Gambler. Non-involvement in raffles is likely predictive because purchasing raffle tickets is often done to support charitable causes rather than to win money.

### Limitations

There are some limitations of this study as it relates to survey research. One potential limitation is the 36.6% response rate attained in the survey. Survey response rates in developed countries have fallen precipitously in recent years; this increases the likelihood that participants differ from non-participants in some important and systematic way (i.e., sampling bias), making the sample non-representative. While this does not always occur [51–53], the risk is always present and tends to increase as a function of the degree of non-response. While we attempted to minimize systematic bias by introducing the study as a survey of ‘health and recreation,’ the response rate for the BGPS was lower than desirable and, as a consequence, generalization of our results should be undertaken with care.

While this study does suffer from a low response rate, it is within the range of response rates achieved in similar studies conducted at about the same time. Prior to the BGPS, three other surveys collected information about gambling and problem gambling in Massachusetts. These included: a module of questions added to the 2013 Behavioral Risk Factor Surveillance System (BRFSS) [3, 4]; an online panel survey funded by the National Center for Responsible Gaming and conducted by the Cambridge Health Alliance Division on Addictions (CHA-DOA) [47]; and an online panel survey funded and carried out by the Massachusetts Council on Compulsive Gambling (MCCG) [48].

The response rate (AAPOR RR4) for the BRFSS—a random digit dial survey—was 39.9% for the combined landline and cell phone sample in Massachusetts (42.6% for landline; 29.5% for cell phone) [54]. The CHA-DOA panel was recruited using Massachusetts members of a GfK Knowledge Panel, an online survey panel that uses an address based sampling (ABS) frame to recruit its members. The household recruitment rate was 16.3% and the response rate was 70.5% [55]. The MCCG online panel did not report a response rate since respondents were self-selected.

Another limitation is that the questionnaire was translated into Spanish but not into other languages. Some communities in Massachusetts have high proportions of adults with no or limited English language abilities. By not providing surveys in additional languages, we were unable to include such individuals in our sample. However, it is our belief that alternate research strategies are desirable to fully explore the role of gambling in a variety of small but important cultural communities in Massachusetts, including Asians and South Asians as well as immigrant and refugee communities.

The small number of respondents in several subgroups in the sample also resulted in estimates with large confidence intervals. These estimates are less reliable. Finally, it is important to emphasize that the BGPS is a cross-sectional ‘snapshot’ of gambling behaviors at a single point in time. This limits our ability to draw any causal conclusions from associations reported between gambling participation, gambling problems, and other variables in Massachusetts.

## Conclusions

There are no marked differences in the health and mental health status of Recreational Gamblers versus Non-Gamblers. While it is true that having drug or alcohol problems was correlated to recreational gambling in the multivariate logistic regression analysis, it was the weakest of the 13 correlates and the actual percentage reporting such problems was small (0.8% of Non-Gamblers versus 1.8% of Recreational Gamblers). The lack of marked differences in the health and mental health of these two groups implies that intervention efforts to prevent harm from gambling should probably not be directed at gambling generally, as recreational gambling can be viewed as a normative activity not clearly associated with elevated harm. Rather, we believe the focus should be more specific to excessive levels of gambling and/or at-risk gambling. What is notable in this comparison is that the strongest correlate of being a Non-Gambler was having a lower portion of friends and family who gamble regularly, which seems to act as a protective factor.

Discriminating between Recreational and At-Risk Gamblers also shows the importance of social networks in relation to gambling behavior. Indeed, the portion of friends and family gambling regularly was the second strongest discriminator of at-risk gambling. This is further indication that targeting the social networks of At-Risk Gamblers is particularly important in prevention. The difference between Recreational and At-Risk Gamblers also reaffirms the notion that certain demographic groups are well suited for targeted prevention. In addition to males and individuals with lower educational attainment, immigrants and individuals with lower income have a higher risk profile. Poorer health was also implicated in the form of higher rates of mental health problems. Interestingly, the only substance-use variable that was significant in the present analysis was that the non-use of alcohol in the previous 30 days was correlated to being an At-Risk Gambler. This may indicate previous trauma in people with a history of alcohol abuse or who come from a family with alcohol abusers which then manifests in a bimodal distribution of alcohol use [44].

Concerning the gambling formats which most discriminate At-Risk and Problem/Pathological Gamblers from Recreational Gamblers, we find that At-Risk Gamblers were most likely to gamble at casinos, play the instant and daily lottery, gamble online, bet privately, and play bingo and Problem or Pathological Gamblers were most likely to play daily lottery games, gamble at casinos, gamble online, and play instant lottery games. When controlling for number of gambling formats engaged in, only casino gambling and not participating in raffles were significant for At-Risk Gamblers while none of these gambling formats were predictive for Problem/Pathological Gamblers (although private betting and non-involvement in raffles do become predictive). These findings suggest that At-Risk and Problem/Pathological Gamblers typically engage in several different types of gambling. Entering number of gambling formats into the model, however, has significant limitations in illustrating the importance of specific gambling formats since it is a major aspect of being an At-Risk Gambler or a Problem/Pathological Gambler.

Similar to the analyses of at-risk gambling, comparing Recreational to Problem/Pathological Gamblers reaffirms that certain demographic groups merit special targeting for intervention, with most of these groups having been identified in previous analyses: males, Blacks, lower educational attainment, and being born outside of the United States. It is interesting that being born outside of the United States was correlated to problem gambling even when controlling for education and race/ethnicity. This may be because many immigrants to Massachusetts come from countries (e.g., Latin America) where legal forms of gambling are less available [45].

One of the strongest discriminators of being a Problem/Pathological Gambler was the portion of friends and family who regularly gamble. Indeed, having a larger portion of friends and family who are regular gamblers is a strong discriminator of being an At-Risk or Problem/Pathological Gambler and having a lower portion of friends and family who gamble regularly is the strongest correlate of being a Non-Gambler. This suggests that targeting the social networks of At-Risk Gamblers and Problem/Pathological Gamblers ought to be a high priority for prevention efforts in Massachusetts.

While people tend to gravitate to other people with similar interests, longitudinal research has shown that friend and family involvement is an important prospective risk factor for future problem gambling [28, 56–58]. Our results not only reaffirm that gambling participation and support among peer groups and family is connected to increased gambling harm [59, 60] but shows that it is one of its strongest correlates. The mechanism by which this occurs is presumably because having a gambling-involved social network both encourages gambling involvement and normalizes excessive involvement. In the case of family members, it likely also speaks to a shared genetic predisposition to problem gambling, the magnitude of which has been shown to be quite substantial [61–63]. From these analyses, it is clear that: a) gamblers need to be aware of the normalizing effect that their social group has on their own gambling behavior; b) friends and family of regular gamblers need to be aware of the facilitative role they have on that person’s gambling; and c) all gamblers need to be aware that problem gambling (and presumably heavy gambling) has a significant genetic basis and thus they need to be particularly vigilant if they have a positive family history.

## Abbreviations

BGPS: Baseline general population survey
PPGM: Problem and pathological gambling measure
